# CCT α is a novel biomarker for diagnosis of laryngeal squamous cell cancer

**DOI:** 10.1038/s41598-019-47895-x

**Published:** 2019-08-14

**Authors:** Jinpu Yang, Zhuping Zhang, Yin Zhao, Jinzhang Cheng, Chang Zhao, Zonggui Wang

**Affiliations:** 1 Department of Otolaryngology-Head and Neck Surgery, The Second Hospital of Jilin University, 218 Ziqiang Street, Changchun, 130041 China; 2 Department of Otolaryngology-Head and Neck Surgery, The Central Hospital of Wuhan, 26 Shengli Street, Wuhan, 430014 China

**Keywords:** Cancer microenvironment, Oral cancer detection

## Abstract

Choline phosphate-based delivery systems can target the acidic tumor microenvironment. In this study, we set out to evaluate the diagnostic value of Choline phosphate cytidylyltransferase-α (CCTα) in laryngeal squamous cell cancer (LSCC). The expression of CCTα was detected using immunohistochemistry in 50 LSCC patients’ tissues and 16 vocal polyps as control group. Then, clinical data was collected and we used receiver operating characteristic curve (ROC) to estimate the potential of CCTα as diagnostic biomarker. We found CCTα levels to be significantly high in the tissues derived from LSCC patients, (*p* < 0.001). Further, we observed a positive correlation of CCTα with tumor size (*p* < 0.001), TNM stage (*p* < 0.001), lymph node metastasis (*p* < 0.001) as well as the grade of LSCC malignancy (*p* < 0.001). Furthermore, AUC was determined to be 0.939 by ROC, and the optimal cutoff value 3.100, with 76.0% sensitivity and 100% specificity. We also found an epigenetic basis of CCTα over-expression in LSCC tissues with significantly reduced methylation of CCTα in LSCC tissues, compared to vocal polyps (*p* < 0.001). These results support epigenetically-induced over-expression of CCTα as a potential diagnostic marker for LSCC.

## Introduction

Laryngeal cancer accounts for 2–5% of new malignancies worldwide^1^. Approximately, 12410 new cases of laryngeal cancer are estimated to be reported in the United States, as reported for 2019 recently^2^ and in China, the incidence of laryngeal cancer is quite alarming^3,4^. Laryngeal squamous cell carcinoma (LSCC) is the major malignant tumor of the larynx, accounting for 85–90% of all laryngeal tumors^1^. The progression of LSCC is a complex and not completely understood^5^. Despite tremendous therapeutic advances in recent decades, improvements in the patients’ 5-year survival rate are still minor^2,5^, which could be probably attributed to late-stage diagnosis and other complex factors.

Phospholipids, being prominent in cell membranes, play an important role in cell division by supplying membrane components. Further, induction of phosphatidyl choline biosynthesis is critical for cell proliferation^6^. Choline phosphate cytidylyltransferase-α (CCTα) is involved in phosphatidyl choline synthesis^7^. It was reported that CCTα may be a promising biomarker for few other cancers^8^. However, the diagnostic role of CCTα in LSCC, if any, has never been reported. We, therefore, designed this study to evaluate CCTα in LSCC through immunohistochemistry (IHC) and to evaluate the association of CCTα with clinicopathology of LSCC patients. Meanwhile, the epigenetic basis of differential expression of CCTα was also determined to complete our understanding of how CCTα expression might differ between control populations and LSCC patients.

## Materials and Methods

### Ethical considerations

Approval for this retrospective analysis was obtained from the institutional research ethics board of the Jilin University prior to enrolment (1106/2015). All the methods were performed in accordance with the relevant guidelines and regulations.

### Patient characteristics

50 blocks of paraffin-embedded LSCC samples were obtained from the Department of Otolaryngology, Head and Neck Surgery, The Second Hospital of Jilin University, operated and treated in 2009–2012. 16 vocal polyp blocks served as control. Informed consent was obtained from all patients. Patients with recurrence or a previous history of cancer were excluded. This study comprised of 40 male and 10 female patients with an average age of 59.8 years (age range: 44–79 years). Stage categories were based on TNM Classification of Malignant Tumours.

### Immunohistochemistry

Specimens of laryngeal carcinomas and benign lesions were removed and fixed in 4% paraformaldehyde (PFA) in phosphate-buffered saline (PBS) overnight at 4 °C, embedded in paraffin and sectioned at 4 μm. Deparaffiisation, hydration and epitope demasking were carried out with a microwave antigen retrieval procedure in sodium citrate buffer for 5 min. Then sections were treated with 3% H_2_O_2_ for 20 min to quench endogenous peroxidase activity and blocked with 5% bull serum albumin (BSA) for 20 min. The primary antibody used were goat anti-rabbit anti-CCTα (1:50, ab109263, Abcam, Cambridge, UK). Slides stained with primary antibodies were incubated at 4 °C overnight. Then the slides were incubated with an anti-rabbit IgG antibody at room temperature for 30 minutes. The binding of the primary antibody to the sections was visualized by using DAB Substrate-Chromogen Solution (Dako Cytomation, Carpinteria, CA, USA), the sections were then counterstained with hematoxylin (Beyotime Biotechnology).

### Evaluation of immunohistochemical reaction

Two pathologists, blinded to the study, carried out the evaluation independently at x1000 magnification, using Eclipse Ni-U (Nikon, Japan) upright microscope coupled with visual circuit and NIS Elements F (Nikon) software for computer image analysis. The evaluation of CCTα expression was determined with the use of five-point evaluation scale^9^, taking into account the intensity of colour reaction (score 0–3) and percentage amount of positive cancer cells in a given specimen (score 0–4). The final result was the product of scores obtained for the evaluation of both parameters and values from 0 to 12 were considered^9^.

### Bisulfite conversion of FFPE samples

To quantitate the differential methylation of FFPE samples, we used the FFPE Bisulfite conversion kit from Active Motif (Shanghai, China) and performed the analyses exactly as recommended by the manufacturer’s instructions. Primer design for evaluating CCTα-specific methylation was done using published method^10^.

### Statistical analysis

All data were analyzed with SPSS statistical software, version 13.0 (SPSS, Inc, Chicago, IL). To evaluate the relationship between the intensity of CCTα expression and LSCC grade, Mann-whitney U test and Kruskal-wallis test were used. And Fisher’s exact test was used to determine the association between CCTα expression level and clinical and pathological factors. In all analyses, values were considered significant at P-value < 0.05.

## Results

### Evaluation of CCTα in LSCC tissues

**Figure 1:**
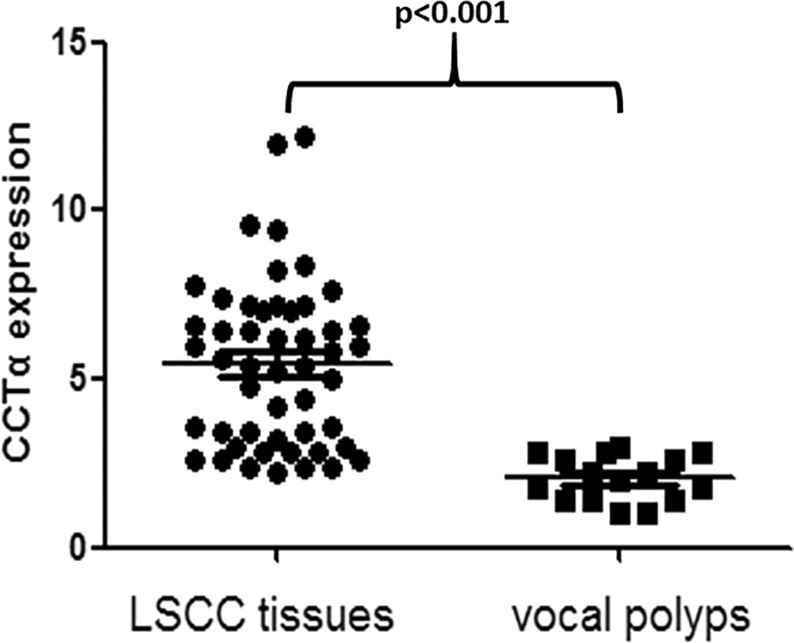
Detection of expression levels of CCTα in LSCC tissues and vocal polyps. The expression of CCTα in LSCC tissues was significantly higher than in controls (*p* < 0.001).

We started the investigation by detecting the differential expression of CCTα in LSCC patients, compared to controls. For this, we analyzed the expression of CCTα by Immunohistochemistry in LSCC tissues and vocal polyps. As seen in Fig. 1, CCTα was significantly up-regulated in tissues derived from LSCC patients, as compared to vocal polyps, the controls (*p* < 0.001).

### CCTα and clinicopathological characteristics

Next, we assessed the correlation between CCTα expression and the clinicopathological characteristics, using Fisher’s exact test. As evident from the data presented in Table 1, CCTα significantly correlated with tumor size (*P* = **0.013**), TNM stage (*P* = **0.000**) and lymph node metastasis (*P* = **0.029**). A majority of high CCTα expressing tumors (22 of 38) corresponded to T3-T4 while a majority of low CCTα expressing tumors (10 of 12) corresponded to T1-T2. For the TNM stage, a majority of high CCTα expressing tumors (27 of 38) correlated with advance stage III-IV tumors while a majority of low CCTα expressing tumors (11 of 12) correlated with low grade stage I-II tumors. Similarly, while low CCTα expressing tumors had no lymph node metastasis, majority of high CCTα expressing tumors were associated with lymph node metastasis. However, we did not find any correlation between CCTα and gender (*p* = 0.934) or age (*p* = 0.631).

**Table 1:** Relationship between CCTα expression and clinicopathological characteristics in LSCC.

Characteristic	Cases	CCTα expression		x2	*p*-values
		Low N = 12	High N = 38		
Gender					
Male	40	9	31	0.007	0.934
Female	10	3	7		
Age (years)					
≤60	28	6	22	0.231	0.631
>60	22	6	16		
Tumor size					
T1–T2	26	10	16	6.211	**0.013**
T3–T4	24	2	22		
TNM stage					
Stage I/II	22	11	11	14.560	**0.000**
Stage III/IV	28	1	27		
Lymph nodes					
N0	28	10	18	4.788	**0.029**
N1–N3	22	2	20		

Expression intensity: low, 0–3; high, 3–12.

Significant differences for the chi-square test are indicated in bold.

### Expression of CCTα in LSCC with different degrees of differentiation

**Figure 2:**
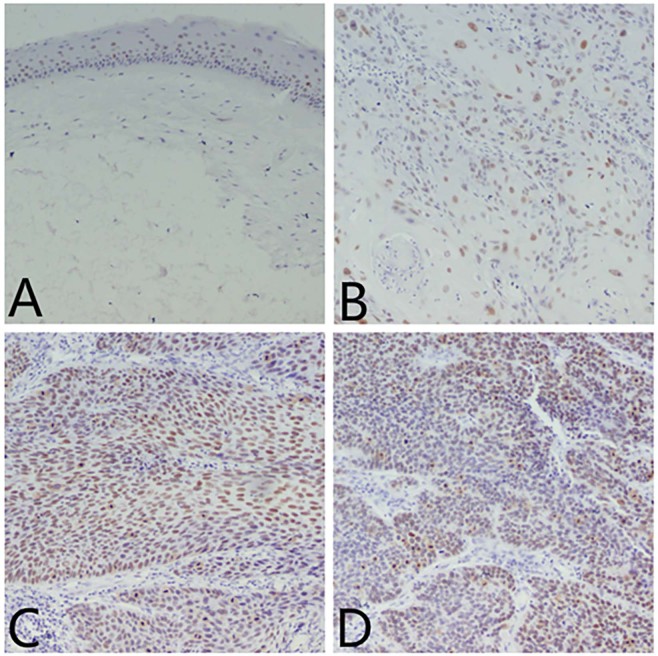
Positive immunohistochemical reaction (brown nuclei) indicating CCTα antigen expression in laryngeal benign lesions and in different histological grades of LSCC. CCTα expression - benign lesion (**A**) and LSCC ((**B**) Grade 1, (**C**) Grade 2 and (**D**) Grade 3).

**Figure 3:**
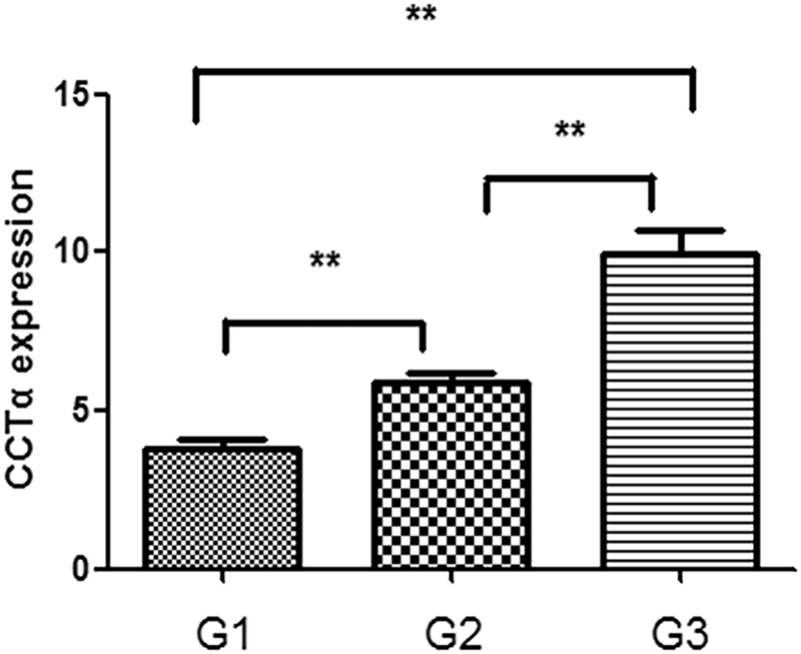
Correlation of expression of CCTα with the grade of LSCC malignancy. **p < 0.01.

IHC revealed nuclear expression of CCTα in LSCC (Fig. 2) in histopathological specimens. The higher the grade of the analyzed LSCC, the greater level of expression of CCTα marker, as evident by brown staining. Grade 1 had the lowest expression among LSCC samples (Fig. 2B), even though it was higher than the control (Fig. 2A). Grades 2 (Fig. 2C) and 3 (Fig. 2D) had substantially more CCTα expression than Grade 1, indicating that CCTα expression correlated with increasing grade of LSCC. Additionally, CCTα was found to be expressed statistically differently among the three grades tested (p < 0.01) (Fig. 3). Combined, the results presented in Figs 2 and 3 support the major conclusion that CCTα expression increases with increasing tumor grade of LSCC.

### CCTα as a diagnostic biomarker

**Figure 4:**
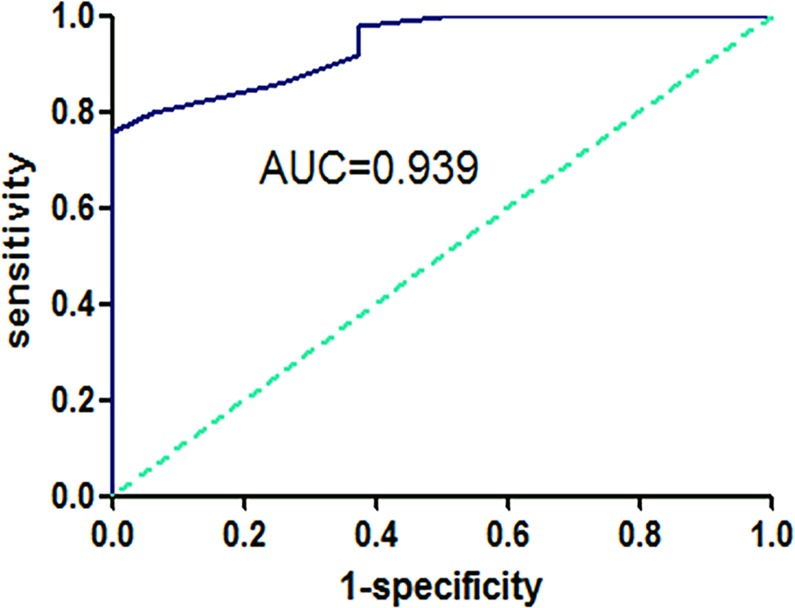
Diagnostic value of CCTα via ROC which had a AUC of 0.939, combining with a sensitivity of 76.0% and a specificity of 100.0%.

We established ROC curve for the estimation of diagnostic value of CCTα, if any, for LSCC (Fig. 4). The AUC value was determined to be 0.939, with 76.0% sensitivity and 100% specificity and a 3.100 cutoff value. This observation supports an interpretation that CCTα is a potential biomarker for differentiating LSCC patients from the controls.

### Methylation of CCTα in LSCC samples

**Figure 5:**
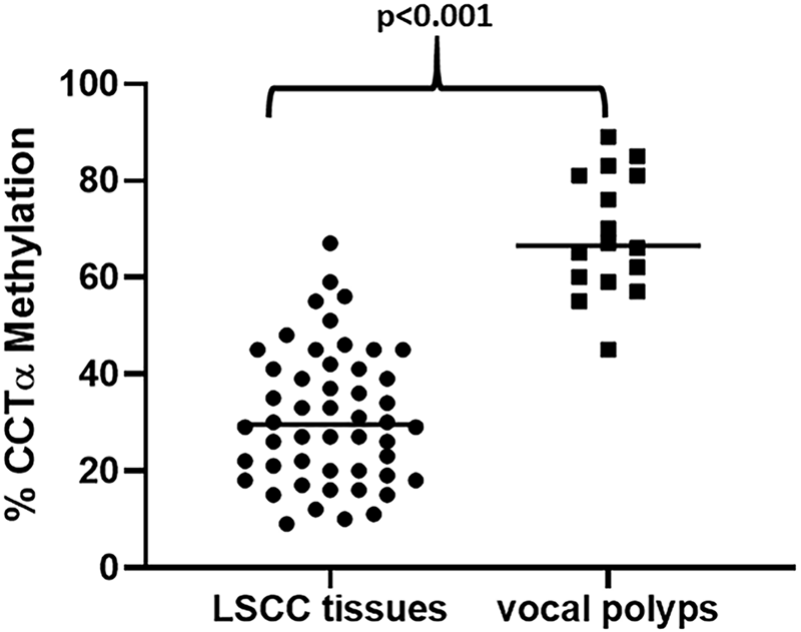
Comparative percentage of CCTα methylation in control (vocal polyps) Vs. tissues from LSCC patients. FFPE samples were used for the analyses. The methylation of CCTα in LSCC tissues was significantly lower than in controls (*p* < 0.001).

Inorder to assess the basis of increased expression of CCTα in LSCC, we performed CCTα-specific methylation evaluation and found that the methylation of CCTα was significantly reduced in LSCC samples (Fig. 5), which may define the underlying cause of increased CCTα expression. It is well known that reduced methylation correlates with increased expression of genes^11^ and therefore our methylation analysis provides a direct evidence supporting epigenetic regulation of CCTα in tumor microenvironment.

## Discussion

LSCC is the predominant laryngeal cancer characterized by long asymptomatic latency and poor prognosis. There is an urgent need for finding and validating novel biomarkers to aid in the time diagnosis of patients. A number of bio-markers, such as cell motility protein 3 (ELMO3)^12^, minichromosome maintenance proteins (MCM)^9^, nucleus protein Ki-67^9^, cytokeratin-18 fragment (CK18)^13^ have been proposed for LSCC by different researchers, however, there is almost no clinical data to verify their possible utility in clinics.

CCTα is a rate-limiting enzyme involved in the biosynthesis of phosphatidyl choline that localizes to the nucleus^7,8,14^. It is involved in the formation of nuclear membrane phospholipid bilayer and the nucleoplasmic reticulum^15^, and is critical for cell viability and embryonic development^15,16^. CCTα plays a role in Ras-transformed cells’ anchorage-independent growth^17^; apoptosis resistance^18^; cell proliferation^19^ and synthesis of phosphatidyl choline^20^, all of which have connection with cancer.

Previous reports have demonstrated that CCTα is another nuclear antigen recognized by 8F1, which happens to be a commonly used monoclonal antibody for the evaluation of ERCC1 expression^8^. However, there is no information on the diagnostic and prognostic value of CCTα, particularly in LSCC patients. To fill the void in our understanding and knowledge, we undertook this study and compared the expression of CCTα in tissues derived from LSCC patients representing different grades. As a control, we used the benign lesions from vocal polyps. Our results speak for themselves as a very clear difference in expression of CCTα was found in the LSCC patients, when compared with controls. Interestingly, increased CCTα expression was noted in higher grade LSCC patients, as compared to low grade LSCC patients, which in itself supports a possible use of CCTα in differential diagnosis of LSCC.

Further, we elucidated the possible mechanism of up-regulation of CCTα in LSCC patients. In recent years, there has been a push towards a better understanding of epigenetic regulations that control cancer^21^. A survey of literature revealed that a few tumor suppressors have recently been reported to epigenetically silenced in LSCC^22,23^. Looks like methylation is the major epigenetic mechanism for the regulation of genes in LSCC^22,23^. We, therefore, evaluated methylation of CCTα in our samples and found significantly reduced methylation of CCTα in LSCC patients which can surely lead to its over-expression. Our results bear resemblance to the report on *TREX2* in laryngeal cancer recently that also correlated reduced methylation with increased gene and protein expression^24^. These results provide a new direction to the research on LSCC and need to be pursued further.

Based on the results presented here, we conclude that the methylation of CCTα is reduced, leading to its over-expression in LSCC patients. Ours is the first report on epigenetic regulation of CCTα in LSCC and its possible diagnostic importance. We argue that these interesting findings need to be further corroborated in larger clinical studies with a focus on mechanistic aspects and the possible therapeutic targeting of CCTα.

## Data Availability

All the data collected for this study has been reported here.
