# Author Correction: CCT α is a novel biomarker for diagnosis of laryngeal squamous cell cancer

**DOI:** 10.1038/s41598-020-76737-4

**Published:** 2020-11-20

**Authors:** Jingpu Yang, Zhuping Zhang, Yin Zhao, Jinzhang Cheng, Chang Zhao, Zonggui Wang

**Affiliations:** 1 Department of Otolaryngology-Head and Neck Surgery, The Second Hospital of Jilin University, 218 Ziqiang Street, Changchun, 130041 China; 2 Department of Otolaryngology-Head and Neck Surgery, The Central Hospital of Wuhan, 26 Shengli Street, Wuhan, 430014 China

Correction to: *Scientific Reports* 10.1038/s41598-019-47895-x, published online 14 August 2019

The original version of this Article contained a typographical error in the spelling of the author Jingpu Yang, which was incorrectly given as Jinpu Yang. This has now been corrected in the PDF and HTML versions of the Article.

